# Supramaximal resection in gliomas: a narrative review of surgical techniques, clinical outcomes, and ethical considerations

**DOI:** 10.1097/MS9.0000000000005140

**Published:** 2026-05-20

**Authors:** Tirath Patel, Hamza Y. Ibrahim, Muhammad F. Mukhtar, Hira Tariq, Kainat Younis, Sana Sohrab, Marriyum R. Khan, Bhumi D. Patel, Nikhilesh Anand

**Affiliations:** a Department of Neurosurgery, Trinity Medical Sciences University School of Medicine, Kingstown, Saint Vincent and the Grenadines; b Department of Surgery, Jinnah Medical and Dental College, Karachi, Pakistan; c Department of Surgery, Azad Jammu Kashmir Medical College, Muzaffarabad, Pakistan; d Department of Surgery, Dow University of Health Sciences, Karachi, Pakistan; e Department of Surgery, Jinnah Sindh Medical University, Karachi, Pakistan; f Department of Surgery, Khyber Medical University, Khyber Pakhtunkhwa, Pakistan; g Department of Surgery, Windsor University School of Medicine, Cayon, Saint Kitts and Nevis; h Department of Medical Education, University of Texas Rio Grande Valley, Edinburg, TX, USA

**Keywords:** fluorescence-guided surgery, glioma, intraoperative MRI, supramaximal resection (SMR), tractography

## Abstract

**Background:**

Supramaximal resection (SMR) represents an evolving frontier in glioma surgery, extending tumor removal beyond conventional contrast-enhancing margins on magnetic resonance imaging (MRI) to include infiltrative, non-contrast-enhancing regions. Rooted in the understanding that gliomas spread microscopically beyond visible boundaries, SMR aims not only to delay recurrence but also to meaningfully prolong survival while preserving neurological integrity.

**Objective:**

This narrative review explores the conceptual foundations, surgical innovations, clinical outcomes, and ethical dimensions surrounding SMR in the management of diffuse gliomas.

**Methods:**

A structured review of the literature published between 2005 and 2025 was conducted across major medical databases. Search terms included “supramaximal resection,” “supratotal resection,” “glioma,” “5-ALA,” “intraoperative MRI,” “direct electrical stimulation,” “tractography,” and “connectomics.” Clinical trials, prospective and retrospective cohorts, and systematic reviews were synthesized to evaluate oncological benefits, functional outcomes, and practical feasibility.

**Results:**

Although definitions of SMR remain heterogeneous, advances in awake craniotomy, cortical and subcortical mapping, intraoperative MRI, fluorescence-guided surgery, and connectome-informed planning have redefined the boundaries of safe resection. Emerging evidence, primarily levels II–III, suggests that extending resection into FLAIR abnormalities beyond the contrast-enhancing tumor may improve overall and progression-free survival, particularly in selected high-grade gliomas. Importantly, functional mapping techniques allow surgeons to pursue oncologic maximization without disproportionately increasing permanent neurological deficits.

**Conclusion:**

SMR reflects a shift from purely anatomical resection toward function-guided precision surgery. While promising, its adoption requires standardized definitions, prospective validation, and careful shared decision-making. Ultimately, the goal of SMR is not only longer survival but survival lived with preserved cognition, independence, and dignity.

## Introduction

Supramaximal resection (SMR) is defined as the surgical resection of glioma tissue beyond the contrast-enhancing tumor margins seen on contrast-enhanced T1-weighted magnetic resonance imaging (MRI). The procedure often involves resection of non-contrast-enhancing regions visualized on T2 fluid-attenuated inversion recovery (FLAIR) imaging to achieve maximal tumor clearance while ensuring the preservation of neurologic function^[^1^]^. Surgical management of gliomas has evolved over the years from simple biopsy to subtotal resection, and eventually to gross total resection (GTR), and is increasingly moving toward SMR, reflecting advances in imaging, surgical expertise, and understanding of tumor biology^[^2^]^. This review aims to provide a critical appraisal of SMR in gliomas, covering its definition, history, and current evidence regarding its effect on patient outcomes.

## Conceptual framework

### Definition variability

The definition of SMR in gliomas is highly heterogeneous across the literature. Some references categorically define SMR as resection beyond the contrast-enhancing (CE) boundary on T1-weighted MRI, while others focus on resection of the non-contrast-enhancing (NCE) areas, particularly those visualized as hyperintense on T2-FLAIR imaging. This has resulted in a wide range of operational definitions, including volumetric thresholds for resection of NCE areas, total resection of FLAIR abnormalities, and even lobar resections in selected cases^[^1^]^. The newly proposed Response Assessment in Neuro-Oncology resect group classification aims to standardize these definitions by defining SMR as complete resection of CE areas plus extensive resection of NCE areas, typically expressed as ≤5 cm^3^ residual NCE tumor^[^3^]^.

### Imaging correlates of SMR

**Figure 1.**
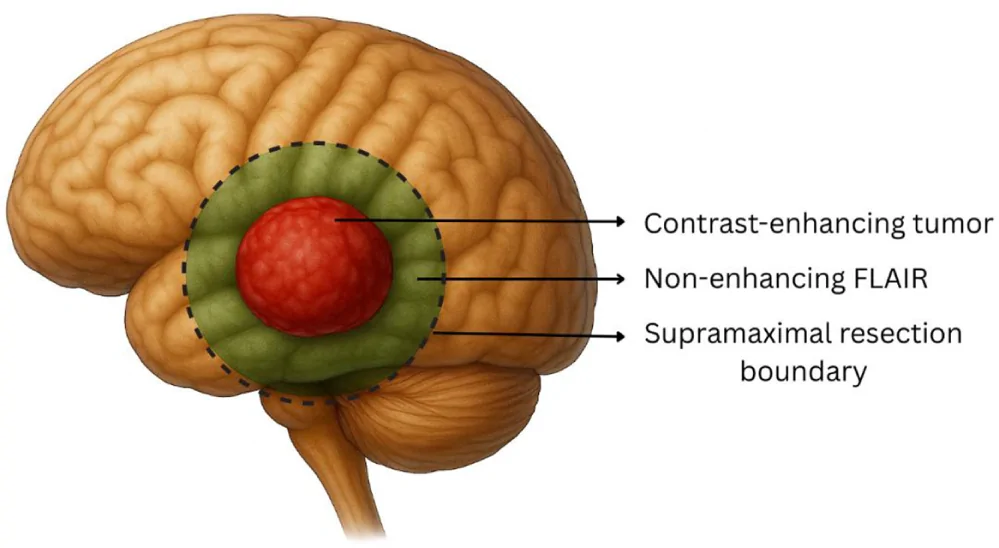
Schematic illustration of tumor margins.

Surgical resection planning and SMR assessment are highly reliant on imaging. Post-contrast T1-weighted MRI delineates the CE tumor core, while T2-FLAIR sequences demonstrate the infiltrative NCE tumor margin. A schematic illustration of contrast-enhancing and non-contrast-enhancing tumor margins, along with SMR boundaries, is shown in Figure 1. Volumetric analysis of both CE and FLAIR abnormalities pre- and postoperatively is now standard in SMR studies, with FLAIR-based extent of resection (EOR) emerging as a stronger predictor of survival than CE-based EOR^[^4^]^. Other newer imaging modalities, such as positron emission tomography and intraoperative fluorescence [e.g., 5-aminolevulinic acid (5-ALA)], are being explored to better delineate resection margins and guide tumor removal^[^1^]^.

### Pathophysiological rationale

The rationale for SMR is based on the pathophysiology of glioma cell migration. Glioma cells invade brain tissue beyond the radiologically apparent tumor margins, often extending up to 10–20 mm into the peritumoral brain parenchyma. Conventional imaging inaccurately assesses the true extent of tumor infiltration, as sub-MRI microscopic disease may be present within normal-appearing MRI compartments. By resecting brain tissue beyond the radiologically defined tumor, SMR may eliminate these infiltrating cells and theoretically reduce recurrence and improve survival, provided that functional neural networks are preserved^[^5^]^.

This manuscript has been made compliant with the TITAN checklist to ensure transparency in the reporting of artificial intelligence (AI)^[^6^]^.

## Methodology

### Literature search strategy

A structured literature search was conducted using PubMed, Scopus, and Web of Science to identify relevant studies. The search covered publications from January 2005 to January 2025, reflecting the period during which SMR gained increasing clinical and technical relevance.

HIGHLIGHTS

- **Redefining glioma surgery**: Supramaximal resection (SMR) extends beyond conventional tumor margins, aiming to reduce recurrence and prolong survival while safeguarding neurological function.
- **Techniques enabling safer SMR**: Awake mapping, intraoperative magnetic resonance imaging, fluorescence-guided surgery (5-aminolevulinic acid), tractography, and connectomics allow surgeons to push the boundaries of safe resection.
- **Clinical outcomes and patient impact**: Evidence shows that SMR can improve survival and seizure control, with many patients regaining quality of life and returning to work.
- **Balancing risks and ethics**: Wider resections must be weighed against the potential for neurological deficits, emphasizing shared decision-making and respect for patient autonomy.
- **Future directions**: Integration of artificial intelligence, connectome-based surgery, and advanced imaging will refine SMR, striving to maximize survival while preserving dignity and cognitive integrity.

The search strategy combined controlled vocabulary and free-text terms. Key search terms included the following: “supramaximal resection,” “supratotal resection,” “glioma,” “high-grade glioma,” “low-grade glioma,” “5-ALA,” “intraoperative MRI,” “direct electrical stimulation,” “tractography,” and “connectome.” Boolean operators (AND/OR) were used to refine search combinations, where appropriate.

To ensure completeness, reference lists of selected key articles and relevant clinical guidelines were manually screened to identify additional pertinent studies not captured in the primary database search.

### Inclusion and exclusion criteria

Studies were included if they described surgical techniques, intraoperative mapping or monitoring strategies, image-guided or visualization adjuncts, or reported functional and oncologic outcomes associated with SMR in gliomas. Eligible study designs included clinical trials, systematic reviews, meta-analyses, and large observational cohort studies published in English. Studies specifically addressing neurosurgical outcomes, survival metrics, and postoperative functional status were prioritized.

Exclusion criteria comprised non-English publications, purely animal or *in vitro* studies (unless deemed highly translational to clinical practice), and small case reports involving fewer than five patients, unless considered historically significant in shaping the concept of SMR.

### Data extraction and synthesis

Titles and abstracts were screened manually, followed by a full-text review of potentially eligible studies. Data extraction was performed independently by two reviewers, focusing on study design, patient population, surgical indications, technical approach, oncologic outcomes, functional outcomes, and the duration of follow-up. Citation management software was used to organize references and remove duplicates.

Given the narrative nature of this review, no formal risk-of-bias assessment or quantitative meta-analysis was conducted. Instead, the synthesis emphasizes practical neurosurgical considerations, comparative strengths and limitations of operative adjuncts, reported survival and functional outcomes, and key ethical considerations relevant to the pursuit of SMR.

## Surgical techniques enabling SMR

Achieving SMR in gliomas requires not merely excellent microsurgical technique but also a combination of modern intraoperative adjuncts to strike a balance between oncologic control and functional outcomes. Several techniques have been developed alongside this approach, including awake craniotomy with mapping, intraoperative imaging and fluorescence guidance, tractography and connectomics, and cortical/subcortical stimulation (SSS). Each plays a distinct role in enabling the neurosurgeon to extend resection beyond what imaging could previously permit without causing significant neurological morbidity.

### Awake craniotomy and intraoperative mapping

Awake craniotomy is the gold standard procedure for maximizing safe resection in eloquent brain regions. The process allows real-time testing of motor, sensory, and higher-order cognitive (e.g., speech, executive functions, and social cognition) functions while the patient remains awake and cooperative. It has been demonstrated in the literature that intraoperative direct electrical stimulation mapping during awake procedures markedly enhances the EOR without increasing permanent deficits^[^7,8^]^.

Recent trends have extended the application of awake mapping beyond traditional motor and language functions to more complex cognitive abilities, such as multitasking, memory retrieval, and decision-making. This shift correlates with the functional connectome model, which emphasizes distributed brain networks rather than localized regions^[^9^]^. In addition, awake mapping has been shown to improve both quality of life and long-term functional outcomes, even when surgery is performed in areas traditionally considered eloquent, such as the insula or supplementary motor area^[^10^]^.

Practically, awake surgery is resource intensive and requires specialized anesthesia teams, psychological preparation, and sophisticated intraoperative neurophysiological monitoring. When effectively implemented, however, it allows the surgeon to extend beyond radiological tumor margins without violating functional boundaries, which is one of the central principles of SMR.

### Intraoperative MRI and fluorescence guidance

Despite careful microsurgical technique, intraoperative landmarks may be insufficient to guide SMR because gliomas extend beyond visible boundaries. Intraoperative MRI (iMRI) has therefore emerged as a complementary tool. It provides real-time imaging and allows the surgeon to assess residual tumor volumes and modify the surgical plan immediately. Randomized controlled trials (level I) have demonstrated that intraoperative MRI improves the EOR and progression-free survival in high-grade gliomas^[^11^]^. Disadvantages include high cost, prolonged operative time, the need for MRI-compatible equipment, and additional operating room infrastructure.

Another method for visualizing tumor margins is fluorescence-guided surgery (FGS), particularly using 5-ALA, which accumulates in tumor cells and emits fluorescence under blue light, enabling the identification of even microscopic tumor infiltration. A landmark randomized controlled trial showed that 5-ALA FGS doubled the proportion of complete resections compared with conventional microsurgery and translated into improved 6-month progression-free survival^[^12^]^.

New fluorophores, such as fluorescein sodium and indocyanine green, as well as tumor-specific molecular probes, are expanding the armamentarium. The advantages of combining FGS and iMRI are complementary: metabolic visualization and anatomical precision on MRI^[^13^]^. These technologies enable SMR to extend resection beyond the contrast-enhancing core into peritumoral infiltrative areas with minimal collateral injury.

### Tractography and connectomics

Anatomical boundaries alone are no longer sufficient to guide SMR in diffuse gliomas, which spread along white matter tracts. Diffusion tensor imaging-based tractography is now an essential preoperative imaging modality, demonstrating major white matter bundles such as the corticospinal tract, arcuate fasciculus, and inferior fronto-occipital fasciculus. Integration of tractography with neuronavigation provides a three-dimensional functional roadmap that helps avoid critical pathways during extensive resections^[^14,15^]^.

However, tractography has limitations, including fiber crossing, false positives, and dependence on diffusion models. More advanced techniques, such as high-definition fiber tractography and constrained spherical deconvolution, have improved accuracy. More importantly, the paradigm of connectomics, mapping large-scale brain networks rather than isolated tracts, has reshaped glioma surgery. Network integrity is increasingly prioritized, and eloquence is recognized as patient specific in SMR^[^16^]^.

Clinically, tractography and connectomics help neurosurgeons move beyond the concept of maximum safe resection toward functional boundary-guided resection. For example, patient-specific language or executive function networks can be mapped and integrated with intraoperative monitoring to enable supratotal excision of tumor tissue without compromising cognitive function^[^17^]^.

### Cortical and subcortical stimulation techniques

Direct cortical stimulation (DCS) and SSS are considered the standard for functional mapping, particularly during awake procedures. Cortical stimulation identifies surface eloquent regions, while SSS delineates critical white matter pathways during tumor resection. Surgeons can dynamically define functional boundaries by integrating stimulation findings with real-time patient feedback as they approach peritumoral areas^[^18^]^.

SMR is particularly facilitated by SSS. Unlike anatomical imaging or preoperative tractography alone, SSS directly assesses the functional integrity of white matter bundles intraoperatively. This technique has demonstrated the remarkable plasticity of the brain, as some so-called eloquent pathways may tolerate limited disruption without long-term impairment, provided resection is halted before critical thresholds are reached^[^19^]^.

In addition, multimodal monitoring approaches (electrocorticography, motor-evoked potentials, and continuous subcortical mapping) enhance safety and reduce false-negative results. These are particularly helpful in repeat surgeries or insular gliomas, where the anatomy may be distorted.

The main limitations include a steep learning curve, the need for patient cooperation, and prolonged operative time. Nevertheless, DCS and SSS remain indispensable in achieving SMR without compromising long-term neurological function.

A summary of various surgical techniques, their clinical utility, strengths, and limitations, is presented in Table 1.

**Table 1:** Surgical adjuncts that permit SMR.

Technique	Clinical utility: how does it assist SMR	Strengths	Limitations	References
Awake craniotomy with mapping	Real-time speech, motor, and cognition testing during resection maximize safe resection in eloquent areas and enhances QoL.	Maximizes safe resection in eloquent areas; improves quality of life (QoL)	Resource intensive; requires patient cooperation	^[^7–10^]^
Intraoperative MRI	Identifies residual tumor during surgery	Improves the EOR and survival; provides real-time updates	Expensive; prolongs operating room (OR) time	^[^11^]^
5-ALA fluorescence guidance	Visualizes microscopic tumor infiltration	Doubles complete resection rates; improves progression-free survival	Needs special light filter; false positives with inflammation	^[^12,13^]^
Tractography/connectomics	Tracks white matter tracts and networks preoperatively	Helps safely extend resections across MRI boundaries	Susceptible to false positives/negatives	^[^14–17^]^
Cortical and subcortical stimulation	Stimulates cortical and white matter pathways during surgery	Gold standard in functional mapping; allows resection up to functional limit	Technically demanding; time-consuming	^[^18,19^]^

## Evidence of clinical outcomes

Glioma surgery has always been about more than just removing a tumor. It is about trying to extend life while not losing the person in the process. Both low- and high-grade gliomas exhibit survival differences that make this balance even more important.

### Levels of evidence and study design considerations

The available evidence on SMR in gliomas is based heavily on retrospective cohorts (level III evidence), prospective observational cohorts (level II), and a few randomized controlled trials of surgical adjuncts over SMR itself (level I).

Direct randomized studies of SMR versus GTR are not yet available due to ethical and methodological limitations. As a result, the bulk of the survival and functional outcome findings on SMR are based on volumetric studies, propensity-matched cohorts, and institutional series.

In this review, therefore, conclusions are placed in context based on the research design, and survival association findings are backed by levels II–III evidence, with technical feasibility being supported by level I evidence on enabling technology, including intraoperative MRI and FGS.

### Low- and high-grade glioma: survival and recurrence patterns

For low-grade gliomas, survival differs by histology. Astrocytomas show a 5-year overall survival of about 58.8%, with a median survival close to 82.8 months. Oligodendrogliomas, in contrast, have better outcomes, with roughly 80.7% alive at 5 years and the median survival not yet reached^[^20^]^. These patients may live for many years, though the risk of malignant transformation requires careful and ongoing monitoring^[^21^]^.

High-grade gliomas, including glioblastomas, are more aggressive. Median survival ranges from 13 to 16 months, with 1-year survival typically below 40% and 5-year survival under 10%^[^22^]^. Adding treatments such as Tumor Treating Fields can extend median survival to around 20.9 months, but outcomes remain limited^[^23^]^. Patients with IDH-mutant tumors tend to live significantly longer, often beyond a decade, when combined with extensive resection^[^24^]^.

Patterns of recurrence also shape surgical strategy. Most glioblastomas recur within 2 cm of the resection cavity, reflecting their infiltrative nature^[^25^]^. Residual tumor cells often lie within peri-tumoral FLAIR regions, which underpins the rationale for supramarginal resection. Removing tissue beyond the visible enhancing tumor can delay local recurrence and reduce purely local relapses^[^26^]^. In cortical glioblastomas, resection of at least 30% of the FLAIR volume has been associated with longer progression-free survival and fewer early recurrences^[^27^]^.

### Functional, cognitive, and quality-of-life outcomes

Functional and cognitive outcomes are central to the discussion. Techniques like awake mapping, intraoperative neurophysiological monitoring, and tractography help preserve language, motor, and executive functions even during extended resections^[^28^]^. Recent studies further broaden this perspective. For example, patients who underwent SMR with functional mapping were found to have higher rates of postoperative seizure control, which directly improved quality of life and reduced long-term antiepileptic burden^[^29^]^. While some patients do experience short-term cognitive decline after surgery, most recover to their preoperative levels over time. In fact, mood symptoms often improve, and seizure freedom becomes more achievable with tailored resections guided by awake mapping^[^30^]^. Beyond clinical recovery, functional outcomes also matter. Many patients can reintegrate socially and professionally, with studies showing higher return-to-work rates after low-grade glioma surgery^[^31^]^.

Details of major clinical studies evaluating the impact of SMR on survival outcomes are summarized in Table 2.

**Table 2:** Summary of major original clinical studies evaluating outcomes of SMR in gliomas.

Study (year)	Study design	Tumor type	Sample size (*n*)	Definition of SMR	Survival benefit	Complication ate	Neurological deficits	Level of evidence
Stummer *et al*^[^12^]^	Randomized controlled trial	High-grade glioma	322	CE resection aided by 5-ALA fluorescence	Improved 6-month PFS vs conventional surgery	Comparable to control	No increase in permanent deficits	I
Certo *et al*^[^4^]^	Prospective cohort	Glioblastoma	56	GTR + resection of FLAIR abnormality (FLAIRectomy)	Significant OS improvement	~18%	~7% persistent deficits	II
Otsuji *et al*^[^32^]^	Retrospective volumetric study	Cortical glioblastoma	60	≥30% resection of peri-tumoral FLAIR region	Prolonged OS and delayed recurrence	~22%	~10% transient deficits	III
Massaad *et al*^[^33^]^	Retrospective cohort	IDH-wild-type GBM	101	Resection beyond CE margins guided by molecular tumor infiltration	Improved OS compared with GTR	Not consistently reported	Not consistently reported	III
Gerritsen *et al*^[^34^]^	Prospective multicenter cohort (protocol)	Glioblastoma	Ongoing	SMR vs maximal safe resection	Survival outcomes pending	Pending	Pending	II
de Leeuw and Vogelbaum^[^35^]^	Systematic review	Diffuse gliomas	18 studies	Variable SMR definitions	Consistent survival association across cohorts	Variable	Variable	II–III

OS, overall survival; PFS, progression-free survival; NR, not reported.

**Figure 2.**
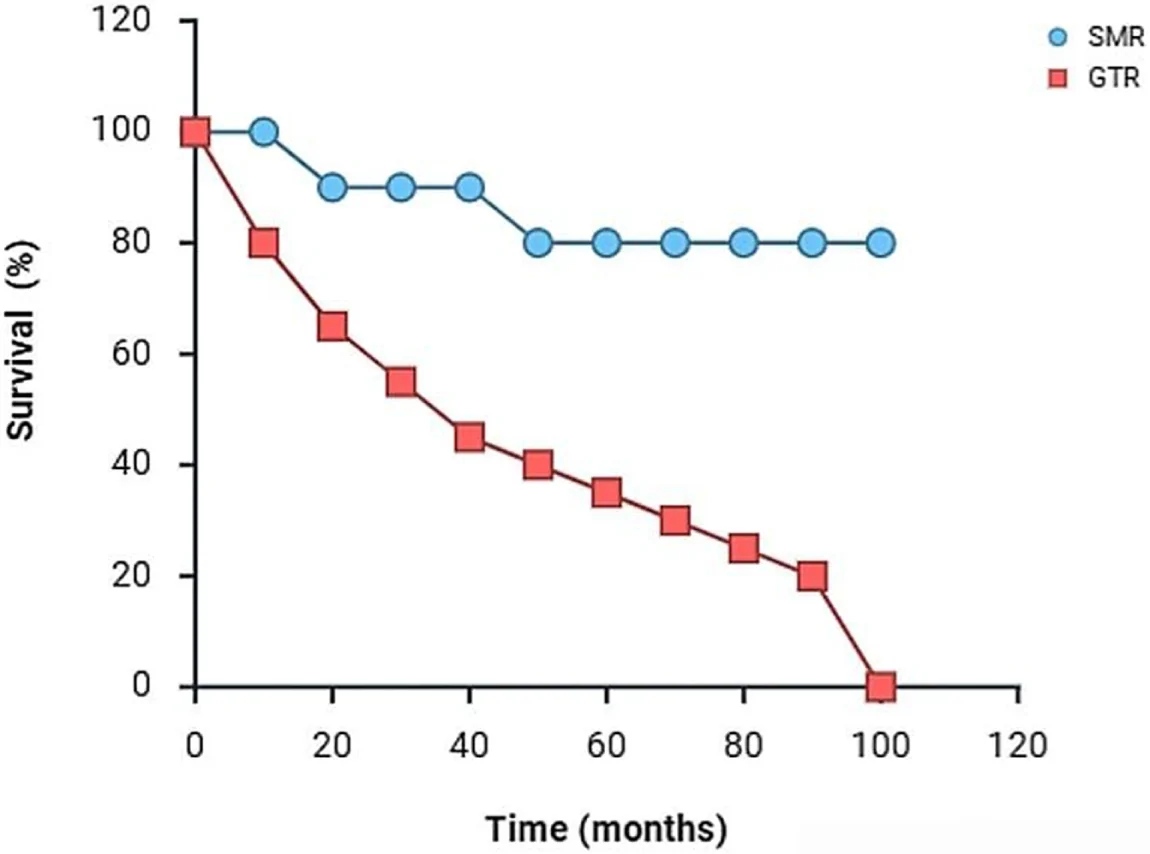
Schematic Kaplan–Meier curves illustrate overall survival for patients undergoing supramaximal resection (SMR) compared with gross total resection (GTR). The data represent synthesized trends from landmark studies, demonstrating improved survival with SMR across glioma subtypes.

A visual synthesis of overall survival trends, comparing SMR and GTR, is presented in Figure 2.

As illustrated in Figure 2, patients undergoing SMR generally demonstrate superior overall survival across multiple glioma subtypes compared with those undergoing GTR, underscoring the oncological benefit of extended resections when functional boundaries are respected.

In conclusion, every surgical decision is more than a technical act; it is a choice made in the shadow of uncertainty and in the service of life. Supramarginal resection may yield longer survival with fewer recurrences, but its first order of evaluation is in the life lived after the operating room. Surgery is not only about taking the tumor out; it is about what kind of life the patient is left with–their dignity, their identity, and their story that carries on.

## Ethical and practical considerations

### Balancing survival and quality of life

Balancing survival and quality of life is an important factor in the treatment of gliomas, especially when addressing radical surgical techniques such as SMR^[^35^]^. The purpose of SMR is to eliminate tumor cells that have infiltrated beyond the CE region, which is potentially favorable for survival but may impair neurological function^[^32^]^. Extensive resections have historically created major functional hazards. However, advances in surgical methods (such as intraoperative mapping and awake surgery), along with a better understanding of tumor borders, are altering the paradigm, allowing more resection without disproportionately increasing neurological impairments^[^2^]^. Despite these developments, the decision to pursue SMR must be carefully considered, since even minor postoperative deficits may compromise patient independence and quality of life^[^36^]^.

### Shared decision-making and ethical balance in SMR

Ethical treatment in neuro-oncology involves shared decision-making (SDM) and specifically applies in the case of SMR, where the possibilities of survival gain must be balanced with potential risks to neurological and cerebral functioning. Recent systematic reviews have shown that organized SDM interventions in neuro-oncology have beneficial effects on patient understanding, decisional conflict, and alignment between treatment decisions and patient values^[^37–39^]^.

In modern SDM models, such as the Three-Talk Model (choice talk, option talk, and decision talk) and the Ottawa Decision Support Framework, clear exposition of risks, advantages, and uncertainties is highlighted. In the operation of glioma, probabilistic gains of survival, probability of impairment of functions, and the effects on the quality of life in the long term are discussed. Hughes *et al* pointed out that such aids as decision-making and structured consultations are especially useful when it comes to multifaceted neurosurgical decision-making, as the evidence is not homogeneous, and the results remain unpredictable^[^37^]^.

SDM has been demonstrated to contribute to the autonomy and psychological health of patients with glioblastoma via the active engagement of patients and caregivers in treatment planning. Musella *et al* also stressed that patients appreciate frank conversations about functional trade-offs and tend to place more importance on independence, cognition, dignity, and survival length^[^38^]^. Likewise, Sorensen von Essen *et al* established that SDM minimizes the level of decisional regret and positively impacts patient satisfaction in high-grade glioma treatment, even when aggressive therapies are sought^[^39^]^.

Ethically, SMR needs a prudent balance of beneficence (oncological maximization) and non-maleficence (harm reduction). Although long-term resections can alleviate recurrence and increase life span, even minor post-operative deficits can have a serious impact on independence and quality of life. SMR requires, to be ethically implemented, therefore, a risk-benefit assessment that is customized, and this is facilitated through functional mapping, connectomic analysis, and clear documentation of patient preferences.

Finally, SMR cannot be viewed as a universal surgical goal but rather as a patient-specific, ethically negotiated approach, which should be implemented only in cases where the limits of functionality are not crossed, and the goals and values of the patient are incorporated into the decision-making process in their entirety.

### Variability in definitions of the EOR

Variability in definitions, particularly regarding the EOR in glioma surgery, poses substantial challenges for clinical translation and consistent patient management. This lack of standardization can result in conflicting interpretations of surgical outcomes and impede effective communication among professionals^[^40^]^. The issue arises because, even with resections that appear equivalent, standard imaging may not adequately depict the infiltrative nature of GBM, leading to inconsistent results^[^33^]^. To address this, a new surgical classification system for glioblastoma has been proposed and validated to provide a more uniform and predictive framework for evaluating resection outcomes. This approach categorizes resections based on absolute residual tumor volume, which has proven to be highly prognostic^[^3^]^.

### Ethical balance

Although SMR may increase survival, it also presents significant ethical and practical challenges. The benefits of extensive tumor removal must be carefully weighed against the risks of neurological impairment and potential effects on quality of life when deciding on SMR. In addition, thorough patient counseling and SDM are crucial, and variability in surgical terminology hinders the translation of study findings into clinical practice.

### Future directions

The existing evidence supporting supramaximal excision in glioma is of low quality and requires more rigorous evaluation before it can be widely adopted as routine practice. Several important future directions have been identified to strengthen the evidence base^[^35^]^. Future directions in glioblastoma precision surgery include key areas such as machine learning (ML), advanced imaging, and the integration of connectome-based techniques to improve patient outcomes^[^41^]^.

### Network-based surgical strategies

The future of glioma surgery lies in a network-based strategy that integrates advanced connectivity analysis with neuromodulation techniques. This paradigm shift aims to improve both oncological and functional outcomes by leveraging the brain’s neuroplastic potential and moving beyond conventional lesion-focused therapies^[^42^]^. Connectome-based surgery represents a significant advancement in neuro-oncology, offering a quantitative and objective method for predicting functional outcomes, tailoring surgical strategies, and ultimately improving the diagnosis, prognosis, and treatment of patients with brain tumors through a more comprehensive understanding of brain networks^[^43^]^. Coordinated research efforts are necessary to overcome current limitations and fully realize the promise of this evolving field^[^42^]^.

### Role of AI and ML

AI and ML play important roles in enhancing surgical precision in neuro-oncology, particularly in determining surgical margins during brain tumor resection. AI assists neurosurgeons in assessing surgical margins and providing real-time diagnostic information and guidance, thereby improving surgical precision and patient outcomes^[^44^]^. ML is being used to integrate data from multiple imaging modalities to distinguish areas of tumor progression from stable regions. This approach can help identify sites where tumors are more likely to recur^[^41^]^. These advancements aim to maximize tumor resection while preserving neurological function, contributing to the concept of safe margins by guiding surgeons on what can be removed without causing unacceptable functional impairment^[^45^]^.

### Clinical trials and emerging therapies

Several prospective clinical trials are currently underway or have recently reported data to advance the surgical management of gliomas^[^46^]^. A blinded prospective study is evaluating stimulated Raman histology for rapid intraoperative diagnosis of CNS tumors. Randomized studies have shown that combining tumor-treating electric fields with temozolomide improves progression-free and overall survival in patients with glioblastoma. Immunotherapy research includes phase I clinical trials using CAR-T cells targeting the EGFR-VIII mutation, which have demonstrated limited efficacy^[^47^]^. Furthermore, emerging techniques such as Raman spectroscopy and optical coherence tomography remain at experimental or early clinical stages, requiring further high-quality research^[^48^]^. These trials are essential for developing evidence-based surgical practices and introducing novel therapies to improve outcomes in patients with gliomas^[^46^]^.

Future developments in glioma surgery will focus on enhancing precision and outcomes through AI-guided resection, connectome-based approaches, and novel diagnostic and therapeutic tools. Validating these advancements, optimizing safe tumor resection, and developing evidence-based strategies to improve patients’ neurological function and survival depend on well-designed prospective trials.

**Figure 3.**
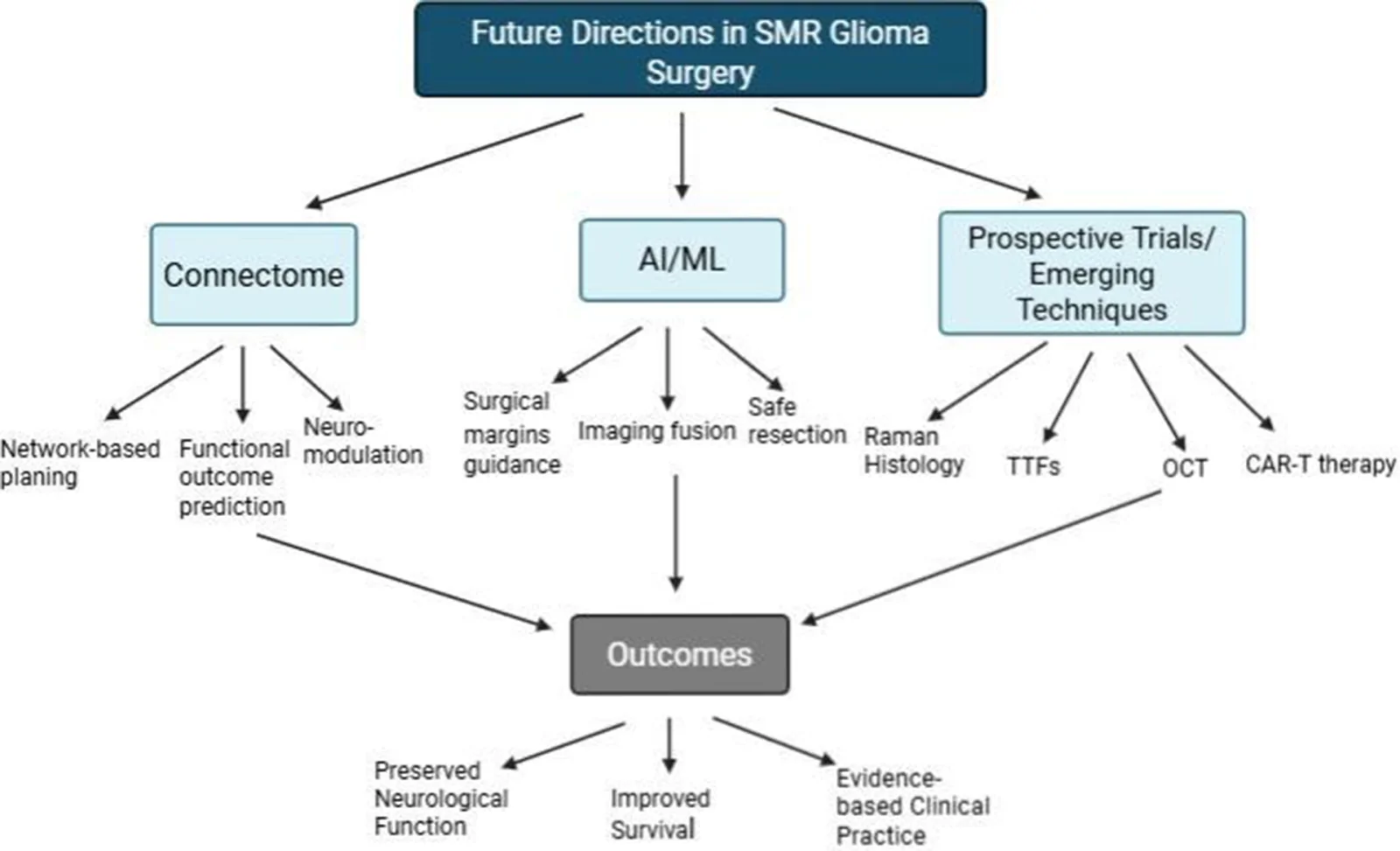
Future directions in SMR glioma surgery: key strategies and anticipated outcomes.

Future trends in SMR, including AI, connectomics, and network-based strategies, are visualized in Figure 3.

## Conclusion

In summary, SMR represents a transformative approach in the management of gliomas, emphasizing the importance of aggressive tumor removal while striving to preserve neurological function. The integration of advanced surgical techniques and imaging modalities has made it possible to navigate the complexities of glioma infiltration, allowing for more comprehensive resections beyond traditional margins. However, variability in definitions and methodologies necessitates a careful and standardized approach in clinical practice to ensure consistent patient outcomes.

Looking ahead, the future of glioma surgery should prioritize the incorporation of cutting-edge technologies and a collaborative framework for patient decision-making. Emphasizing ethical considerations, SDM, and personalized treatment plans will empower patients and enhance their overall experience. As our understanding of SMR continues to evolve, a balanced approach that prioritizes both oncological efficacy and patient quality of life will be essential in delivering optimal care for individuals facing glioma diagnoses.

Notably, existing evidence supporting SMR is largely at levels II–III, highlighting the need for prospective research and standardized outcome reporting to optimize patient selection and ensure ethical practice.

## Data Availability

All data used in this narrative review are publicly available and sourced from previously published studies. No new data were generated for this work. All included articles have been appropriately cited within the manuscript and are available through the references section.

## Research registration unique identifying number (UIN)

The research registration unique identification number (UIN) was not required because it is a narrative review.
